# Using the Standard Deviation of a Region of Interest in an Image to Estimate Camera to Emitter Distance

**DOI:** 10.3390/s120505687

**Published:** 2012-05-03

**Authors:** Angel E. Cano-García, José Luis Lazaro, Arturo Infante, Pedro Fernández, Yamilet Pompa-Chacón, Felipe Espinoza

**Affiliations:** 1 Telecommunication Department, University of Oriente, Av. de las Américas, SN, Santiago de Cuba 90100, Cuba; E-Mails: acano@fie.uo.edu.cu (A.E.C.-G.); ainfante@fie.uo.edu.cu (A.I.); ypompa@fie.uo.edu.cu(Y.P.-C.); 2 Electronics Department, University of Alcalá, Superior Polytechnic School, University Campus, Alcalá de Henares 28871, Madrid, Spain; E-Mails: pedro.fernandez@depeca.uah.es (P.F.); felipe@depeca.uah.es (F.E.)

**Keywords:** standard deviation, distance estimation, infrared, differential method, artificial vision

## Abstract

In this study, a camera to infrared diode (IRED) distance estimation problem was analyzed. The main objective was to define an alternative to measures depth only using the information extracted from pixel grey levels of the IRED image to estimate the distance between the camera and the IRED. In this paper, the standard deviation of the pixel grey level in the region of interest containing the IRED image is proposed as an empirical parameter to define a model for estimating camera to emitter distance. This model includes the camera exposure time, IRED radiant intensity and the distance between the camera and the IRED. An expression for the standard deviation model related to these magnitudes was also derived and calibrated using different images taken under different conditions. From this analysis, we determined the optimum parameters to ensure the best accuracy provided by this alternative. Once the model calibration had been carried out, a differential method to estimate the distance between the camera and the IRED was defined and applied, considering that the camera was aligned with the IRED. The results indicate that this method represents a useful alternative for determining the depth information.

## 1. Introduction

The geometrical camera model uses a mathematical correspondence between image plane coordinates and real word coordinates by modeling the projection of the real world onto the image plane [1,2]. If only a camera is used to estimate the coordinates of a point in space, then the geometrical model will provide two equations; thus, an ill-posed mathematical problem will be obtained. For this reason, an additional camera is used to perform 3-D positioning [3–5].

This problem is known as depth estimation. A general approach to solving this problem is to introduce additional constraints into the mathematical system. These constraints can be obtained from other sensor devices (cameras, laser, *etc*.) [1,6,7].

In addition, the geometrical model only uses the image coordinates as the principal source of information, and image gray level intensities are only used to ensure correspondence among images [8–11].

In references [8–11] it has been demonstrated that only using the pixel gray level intensities of an IRED image, a measurement of depth can be obtained under certain specific conditions, such as:

- Images must be formed by the energy emitted by the IRED. The rejection of background illumination is obtained using an interference filter centered on 940 nm with 10 nm of bandwidth. This implies to using a 940 nm IRED.
- The IRED must be biased with a constant bias current. This guarantees constant IRED radiant intensity.
- The IRED and the camera must be aligned. This means that the radiant intensity as a function of the IRED orientation angle is also constant.

Under these conditions, the distance between the camera and the IRED can be obtained from relative accumulated energy [10] or from the zero-frequency component of the image FFT [11]. Both of the models proposed in [10] and [11] depend on camera exposure time, IRED radiant intensity and distance between the camera and the IRED. They also depend on IRED orientation angle, but this has not yet been taken into account.

Strategically, it would be advantageous to find another parameter and relate it to camera exposure time, IRED radiant intensity and camera to IRED distance. This process would increase the number of constraints extracted from images, thus improve the algorithm in future implementations.

By *strategically*, we mean that, if [10] and [11] are applied together, then each image will provide two equations; however, the problem has three degrees of freedom: the distance between the camera and the IRED, the radiant intensity of the IRED and the IRED orientation angle.

The distance between the camera and the IRED is the main unknown, but in a future implementation the radiant intensity and the IRED orientation angle will need to be estimated or excluded from the final distance estimation alternative.

Regarding the IRED characteristic, the IRED radiant intensity is fixed by the bias current and varies with temperature, material aging, and other factors. Thus, radiant intensity will introduce drift into the distance measurement alternative.

To solve the ill-posed problem, at least one other parameter must be considered to define the final non-geometrical alternative for measuring the distance between the camera and an IRED.

## 2. Background

**Figure 1.**
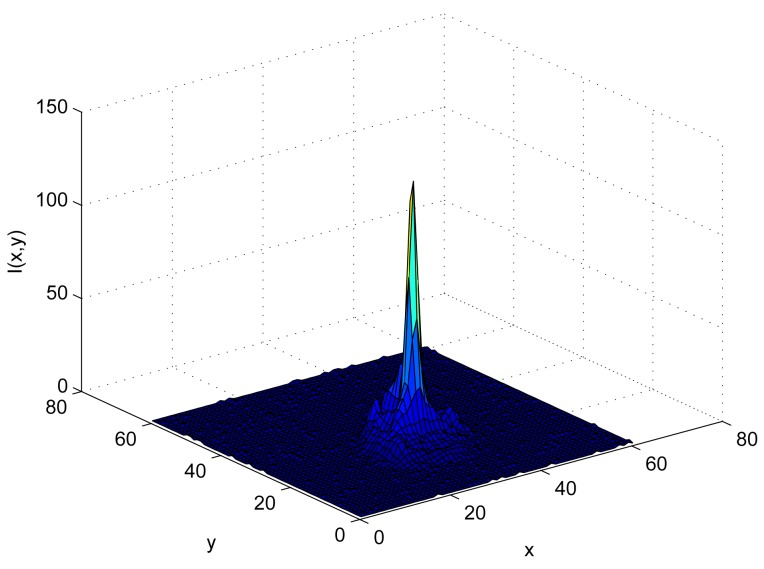
IRED image taken by the camera represented as a 3-D surface.

Figure 1 shows the distribution of the gray level intensity *I*(*x, y*) of an IRED image, with *x* and *y* representing the image rows and columns, respectively.

In the representation shown in Figure 1, the gray level intensity profile of the IRED image was plotted as a 3-D function. When an image of the IRED is captured by the camera, the gray level profile will be a projection in a plane of the 3-D intensity profile of the IRED. Also, in most cases, the 3-D IRED intensities profile can be approximated by a bidimensional Gaussian function [12].

The central peak that is shown in Figure 1 corresponds with the maximum intensity emitted by the source, which in most cases corresponds with the energy emitted in the axial axis of the IRED emission pattern [12].

This representation takes into account the gray level intensities of the image pixels, which were obtained by the energies that fall on sensor surface and were accumulated by the camera during the image capturing process.

Reference [10] proposed using the camera inverse response function [13,14] to obtain a measure of the relative energy accumulated by the camera, in a region of interest containing the IRED image, during the exposure time. This accumulated energy depends on the pixel gray level in the IRED image and decreases with the squared distance between the camera and the IRED.

To estimate the relative accumulated energy, the camera inverse response function must be used. This function establishes a correspondence between pixel gray level intensity at the camera output and the energy accumulated by the camera during the exposure time [13,14]. This energy is the exclusive cause of the light that is emitted by the IRED and falls on the pixel surface.

In [11] only the gray level intensity distribution of the IRED image was used to obtain the FFT, based on the fact that image gray level distribution will change when distance between camera and IRED, exposure time or IRED radiant intensity are changed.

Comparing the parameters proposed in [10] and [11], changes in distance, IRED radiant intensity or camera exposure time produce more evident changes in accumulated energy than the zero-frequency component of image FFT. Nevertheless, in both cases, changes in these magnitudes affect pixel gray level distribution, as reported in [11], and these changes will be evidenced in statistical parameters extracted from images.

Therefore, similar to the procedure followed in [10,11], a statistical parameter is extracted from pixel gray level distribution of IRED images and relate it to the distance between the camera and the IRED, the IRED radiant intensity and camera exposure time.

In this case, the standard deviation of the image gray level intensities that were included in the region of interest and contained the IRED image is proposed as the empirical parameter to be extracted from the IRED image. Note that the standard deviation depends exclusively on the pixel gray level distribution, rather than on the pixel position that is used in projective models [1].

The standard deviation (Σ) provides a measure of the dispersion of image gray level intensities and can be understood as a measure of the power level of the alternating signal component acquired by the camera. Therefore, a relationship would exist between the standard deviation and the camera exposure time, IRED radiant intensity and distance, assuming that the IRED and the camera are aligned.

To use Σ in order to derive an alternative for measuring the distance between the IRED and the camera, a model for the standard deviation must be obtained. In other words, an expression for *F* in Equation (1) must be obtained, bearing in mind that *F* depends on camera exposure time (*t*), the IRED radiant intensity (*I_p_*) and the distance between the camera and the IRED (*d*).

(1) $$\sum =F(t,I_{p},d)$$

To estimate the function *F*, the individual behavior of Σ with *d, t* and *I_p_* were measured.

In all cases, a region of interest containing the IRED image is selected in the processed image. For example, Figure 1 shows a 3-D representation of this region of interest. The region was converted into a vector column-wise or row-wise. There is no difference in the calculation of the standard deviation. Thus, the standard deviation of pixel gray level is obtained by:

(2) $$\sum ={\left(\frac{1}{n-1}\sum_{i=1}^{n}{\left(g_{i}-\bar{g}\right)}^{2}\right)}^{\frac{1}{2}}$$

where *g_i_* represents the gray value of the pixel *i* and $\bar{g}=\frac{1}{n}\sum_{i=1}^{n}g_{i}$ is the mean value of gray level in the image vector.

### 2.1. Behavior of Σ with Camera Exposure Time (t)

To characterize Σ obtained by Equation (2) with camera exposure time, images at a fixed distance with fixed IRED radiant intensity and different camera exposure times were captured.

**Figure 2.**
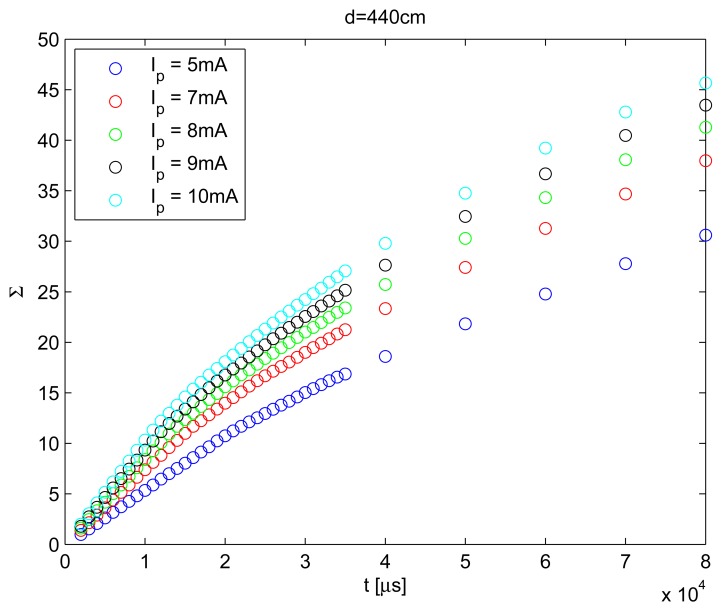
Behavior of Σ with camera exposure time (*t*) obtained for different IRED radiant intensities (*I_p_*) with a distance between the camera and the IRED of 440 cm.

For each condition, 10 images were acquired. The final value for Σ in each condition was the median value over the 10 images acquired. The result of this behavior is shown in Figure 2.

**Figure 3.**
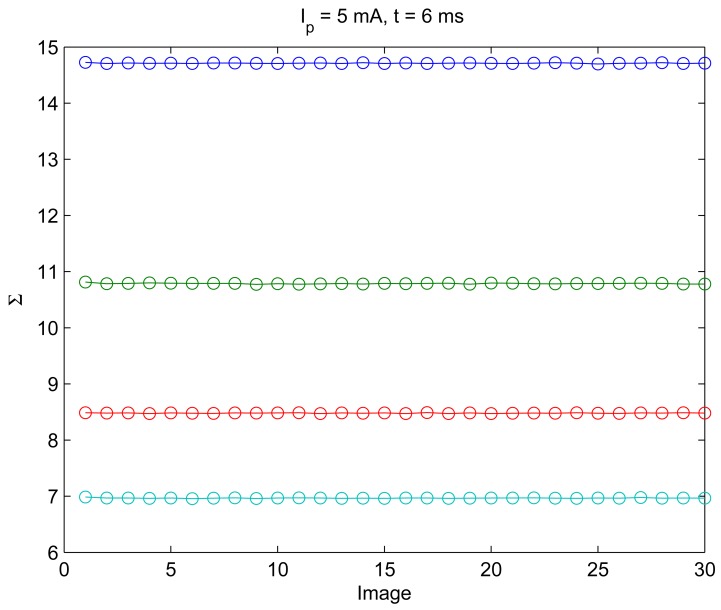
Consistency of the Σ parameter extracted from the IRED images. The maximum dispersion over the 30 images was lower than a 0.5% of the mean value for each considered condition.

The 10 images for each condition were used to perform a statistical model and also ensure the reduction of noise in the behavior of Σ in the model characterization process. Nevertheless, the consistency of the Σ parameter was measured before the behaviors were obtained. Figure 3 shows the result of the measurement of Σ's consistency.

Figure 3 means that in 30 images, the Σ parameter is kept almost constant; specifically the dispersion of the Σ in this experiment was lower than 0.5%. This demonstrates that it is not necessary to perform a more rigorous statistical average to obtain lowest error in the modeling process. That is why only 10 images were used to perform the average to reduce the noise in Σ.

**Figure 4.**
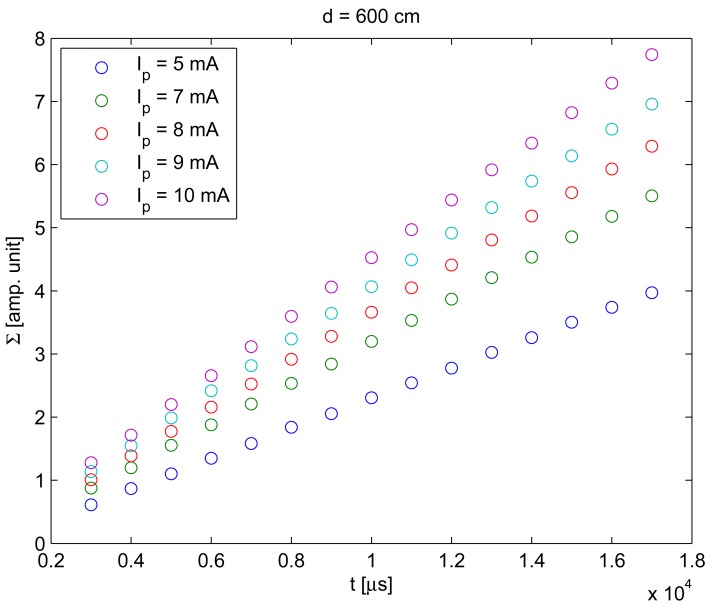
Behavior of Σ with *t* over the range from 2 ms to 18 ms, considering different radiant intensities at a fixed distance between camera and IRED.

As can be seen in Figure 2, total behavior can be modeled with a non-linear relationship with camera exposure time. However, the range of exposure times (*t*) was limited from 2 ms to 18 ms. In Figure 4, the values of Σ over the range of considered exposure times are shown, and this range was used to define the behavior of Σ with *t*.

Under these conditions, Σ can be modeled by a linear function of camera exposure time. Mathematically it can be written as:

(3) $$\sum =\zeta (I_{p})\times \delta (d^{-2})\times (\tau_{1}t+\tau_{2})$$

where *ζ* is the function used to model the behavior of Σ with IRED radiant intensity *I_p_, δ* is the function to model the behavior with distance *d* between the camera and the IRED, and *τ*_1_ and *τ*_2_ are the coefficients to model the linear relationship between *t* and Σ.

The non-linear behavior of Σ with the exposure time shown in Figure 2 could be associated with pixel saturation. For example, when *t* increases, the energy falling on the sensor surface also increases and produces pixel saturation. From an energy point of view, a saturated pixel produces loss of information, because when gray level intensity is used (255 for an 8 bit camera), the recovered energy value is always lower than the real energy value. For this reason, it is advisable to restrict the dynamic range of exposure times to guarantee non-saturated pixels.

### 2.2. Behavior of Σ with IRED Radiant Intensity (I_p_)

**Figure 5.**
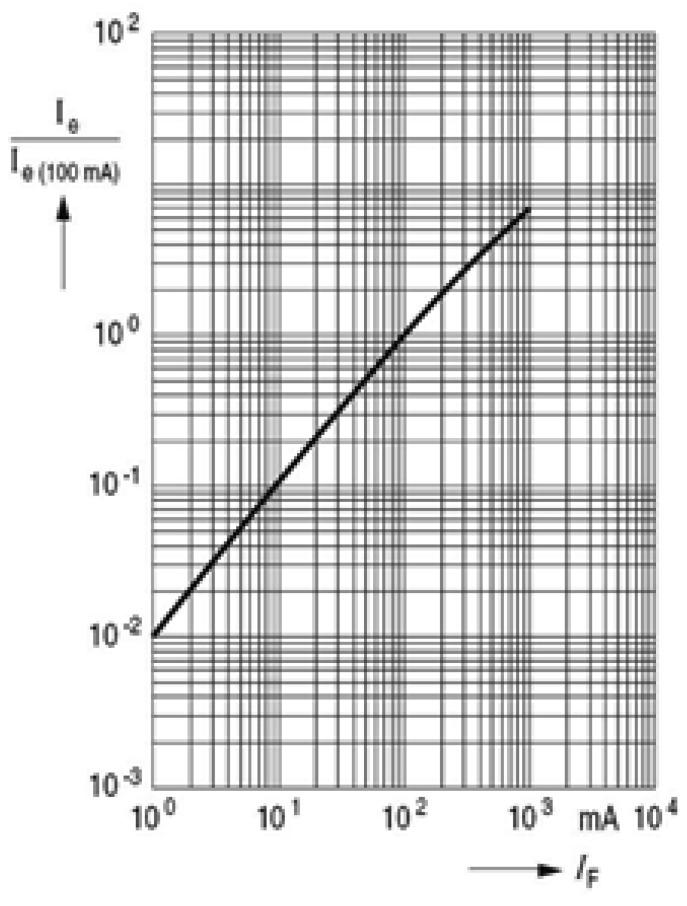
Relationship between IRED radiant intensity and the bias current.

IRED radiant intensity can be controlled by the IRED bias current, as is shown in Figure 5.

Figure 5 shows the behavior of IRED radiant intensity with the bias current. This behavior can be modeled by a linear function. Therefore, changes in IRED radiant intensity can be obtained by changes in IRED bias current.

**Figure 6.**
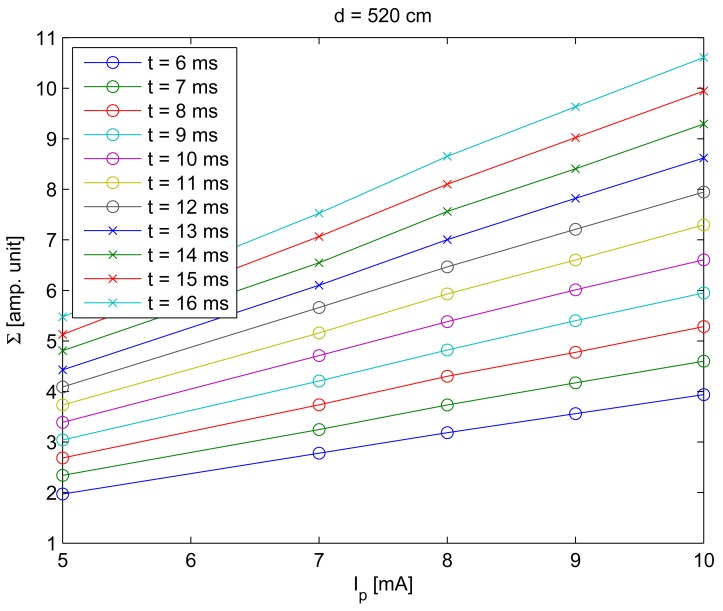
Values of Σ obtained for a distance between the camera and the IRED of 520 cm, for different exposure times (6, 7, … , 16 ms) and for different IRED bias currents.

To characterize the behavior of Σ with the IRED radiant intensity, images captured with different bias currents were used. Figure 6 gives the standard deviation values, calculated by Equation (2) considering different IRED radiant intensities.

Figure 6 was obtained using a fixed distance between the camera and the IRED, ten different exposure times and different IRED bias currents. The behavior of Σ with *I_p_* was considered as a linear function. Thus, the function *ζ* shown in Equation (3) can be written as:

(4) $$\zeta (I_{p})=\rho_{1}I_{p}+\rho_{2}$$

where, *ρ*_1_ and *ρ*_2_ are the coefficients used to model a lineal relationship between Σ and *I_p_*.

### 2.3. Behavior of Σ with the Distance between the Camera and the IRED

**Figure 7.**
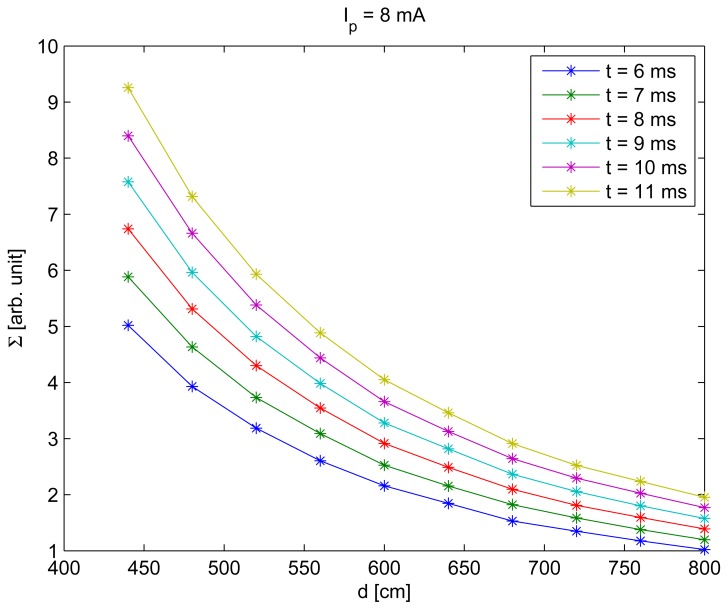
Values of Σ obtained for *I_p_* = 8 mA, with *t* = 6, 7, … , 11 ms and considering distances from 440 to 800 cm.

To include the distance between the camera and the IRED in Equation (1), the behavior of Σ with distance was also measured. The result of this behavior is shown in Figure 7.

To obtain the result shown in Figure 7, exposure times were varied from 6 to 11 ms, the *I_p_* was 8 mA and the distance was varied from 440 to 800 cm. It can be seen in this figure that a relationship exists between the distance and Σ.

**Figure 8.**
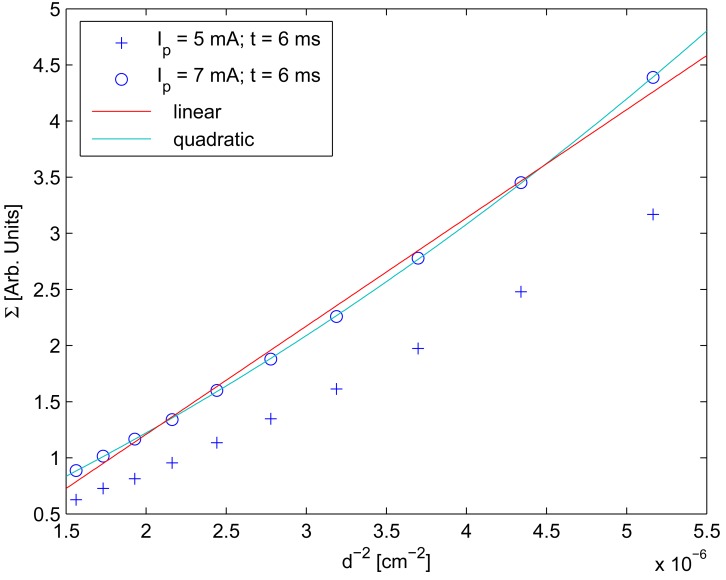
The standard deviation as a function of *d*^−2^ From the behavior of Σ with *d* it can be stated that considering quadratic behavior is more accurate than linear behavior.

However, distance behavior was considered quadratic, rather than linear as in references [10,11]. Figure 8 was generated in order to demonstrate that considering behavior quadratic rather than linear is more accurate.

Finally, the behavior of Σ with the distance between the camera and the IRED were considered as a quadratic function. Therefore, in the Equation (1) the function *δ*(*d*) yields:

(5) $$\delta (D)=\gamma_{1}D^{2}+\gamma_{2}D+\gamma_{3}$$

where, $D=\frac{1}{d^{2}}$ and *γ_i_* with *i* = 1, …, 3 are the coefficients used to model the behavior of Σ with the distance between the camera and the IRED.

## 3. Model of Standard Deviation to Estimate Distance

The behaviors measured and presented in Sections 2.1, 2.2 and 2.3 were integrated into a model that theoretically characterized the standard deviation of pixel gray level in the region of interest containing the IRED image.

Taking into account the Equation (3) and substituting *ζ* for the Equation (4) and *δ* for the Equation (5), Σ can be written as:

(6) $$\sum =(\tau_{1}t+\tau_{2})\times (\rho_{1}I_{p}+\rho_{2})\times (\gamma_{1}D^{2}+\gamma_{2}D+\gamma_{3})$$

After parenthesis elimination, the standard deviation yields:

(7) $$\sum =[\kappa_{1}tI_{p}D^{2}+\kappa_{2}tI_{p}D+\kappa_{3}tI_{p}+\kappa_{4}I_{p}D^{2}+\kappa_{5}I_{p}D+\kappa_{6}I_{p}+\kappa_{7}tD^{2}+\kappa_{8}tD+\kappa_{9}t+\kappa_{10}D^{2}+\kappa_{11}D+\kappa_{12}]$$

where $D=\frac{1}{d^{2}}$ and *κ_i_* with *i* = 1, 2, …, 12 are the model coefficients that can be obtained in a calibration process.

### 3.1. Model Calibration

To calibrate the model proposed in Equation (7), images taken with different radiant intensities, different exposure times and different distances were required. In this case, orientation angles were not taken into account since the camera and the IRED were considered aligned facing one another.

The data used to obtain the values for the model coefficients are summarized in Table 1. Note that IRED radiant intensity are fixed by the IRED bias current.

For each distance, five IRED bias currents were considered. For each distance and IRED bias current, 16 images with different exposure times were used; therefore *N_eqns_* = 192 equations were formed to obtain 12 model coefficients, which are the unknowns.

From each of the equations, the error between the modeled and measured standard deviation can be defined. Thus:

(8) $$\epsilon =\sum_{i=1}^{N_{\text{eqns}}}{\left[\sum_{i}^{\text{measured}}-\sum_{i}^{\text{modeled}}\right]}^{2}$$

where *Σ_modeled_* are obtained by Equation (7) and *Σ_measured_* are extracted from each image by using the Equation (2).

The values for the model coefficients were calculated to minimize the error stated in Equation (8).

**Figure 9.**
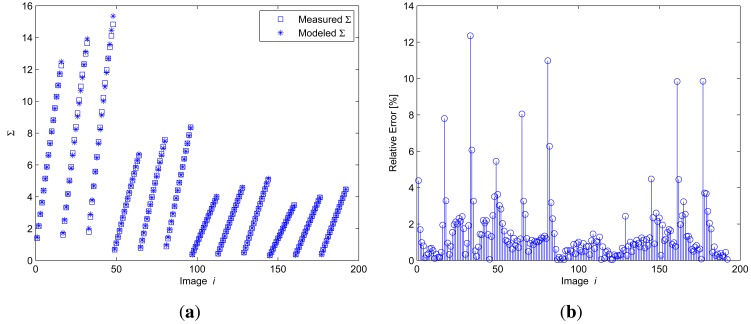
Result of the calibration process.

The coefficients k can be calculated by k = M^+^ × S, where M^+^ = (M^t^ × M)^−1^ × M is the pseudo-inverse matrix of M, which is formed with the calibration data summarized in Table 1 and S is the vector of all measured Σ*_i_*. The results of the calibration process are shown in Figure 9.

In Figure 9(a), the blue squares represent the measured Σ and the asterisks represent the modeled one.

The relative error in the calibration process described, which is shown in Figure 9(b), has peak values lower than 13% for some images. These images, as can be seen in Figure 9(a), corresponded to higher bias currents and higher camera exposure times. However, the mean error was lower than 2%, as is shown in Figure 9(b).

### 3.2. Using the Standard Deviation to Estimate the Distance Between the Camera and the IRED

Once the coefficients had been obtained, the model proposed in Equation (7) could be used to estimate the distance between the camera and the IRED. Similar to references [9-11], a differential methodology was used to estimate the distance.

The differential method used two images captured with different exposure times. Consider two images (*I_j_* and *I_r_*) taken with times *t_j_* and *t_r_*, respectively; ΔΣ is the difference between Σ*_j_* and Σ*_r_*, which are the standard deviation extracted from the images *I_j_* and *I_r_*, respectively. Analytically, ΔΣ would yield:

(9) $$\Delta \sum =\kappa_{1}I_{p}D^{2}\Delta t+\kappa_{2}I_{p}D\Delta t+\kappa_{3}I_{p}\Delta t+\kappa_{7}\Delta tD^{2}+\kappa_{8}\Delta tD+\kappa_{9}\Delta t$$

where Δ*t* = *t_j_* − *t_r_*.

From the Equation (9), the distance can be written as a quadratic function. That is:

(10) $$(\kappa_{1}I_{p}\Delta t+\kappa_{7}\Delta t)\times D^{2}+(\kappa_{2}\Delta tI_{p}+\kappa_{8}\Delta t)\times D+(\kappa_{3}\Delta tI_{p}+\kappa_{9}\Delta t-\Delta \sum )=0$$

Then, the distance estimation can be obtained from the positive real root of Equation (10), considering that $d=\sqrt{\frac{1}{D}}$.

As can be seen from Equation (10), the differential method reduced the number of coefficients used for distance estimation and, as was demonstrated in reference [10], also ensured better performance than the direct distance estimation method.

Another question must be considered; for example, the Equation (10) was defined considering only a Δ*t* extracted from two images. When more images are used for distance estimation, more distance values will be obtained. This means that the number of distance estimation will be the same as the Δ*t* considered in the measurement process. Thus, the problem could be stated as: which time differences must used for distance estimation?

In reference [15], an analysis of error in measurement process was carried out and it was demonstrated that a relationship existed between accuracy in distance estimation and difference of exposure times used in the measurement process. By plotting the distance estimation error, the resulting curve resemble a *bathtub* curve. Furthermore, in reference [15], it was demonstrated that optimum exposure times can be determined in the calibration process.

Using the calibration data summarized in Table 1 and applying the method proposed in reference [15] it is possible to obtain the performance of the model coefficient adjustment to detect the Δ*t* where the lowest error in model fit is obtained. Evidently, the best results in distance estimation will be obtained under those conditions where a best model fit has been obtained.

**Figure 10.**
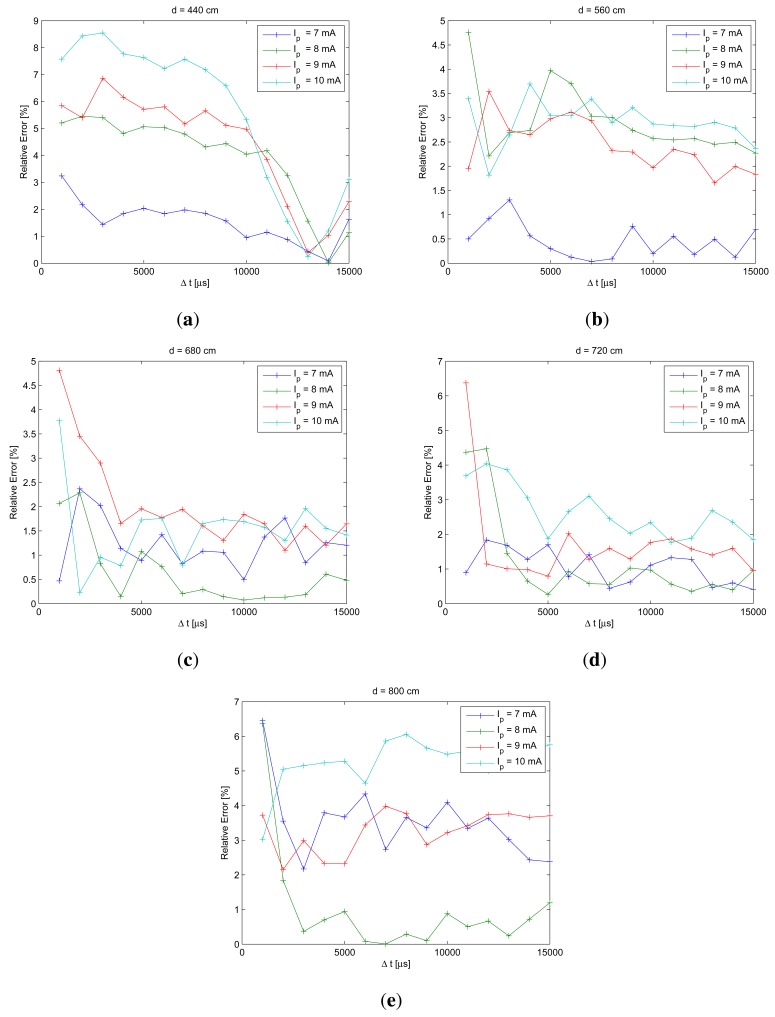
Relative error in the calibration process as a function of exposure time differences. In these figures different distances were considered: (a) 440 cm, (b) 560 cm, (c) 680 cm, (d) 720 cm and (e) 800 cm. From the performance of the calibration process it is possible to obtain the optimum exposure time differences to carry out the distance measurement process. Optimum Δ*t* to carry out the measurement process coincides with Δ*t* where lowest error was obtained in the calibration process.

Once model coefficient had been calculated in the calibration process, the model was written in the differential form to estimate the error in the calibration process. This process was used to evaluate the performance of the model and to detect the values of time differences where lowest error would be obtained for each bias current. Figure 10 shows the results of this analysis.

From Figure 10, the differences of exposure times where best model fit is obtained can be extracted. To generate these figures, *t_r_* = 2 ms and *tj* = 3, 4, …, 17 ms; the exposure time differences would yield: Δ*t* = 1, 2, …, 15 ms. For each IRED bias current, we estimated the optimum exposure time difference for each calibration distance. The obtained values were: 1, 2, 3, 5, 7, 10, 11, 12, 13, 14 and 15 ms. Subsequently, 13 ms was selected as the final optimum exposure time difference to be used in the measurement process, because it was the most frequently repeated value for all considered bias currents and distances.

In addition, in Figure 10 it can be seen that relative error in the calibration process for most distances was lower than 4%. The relative error curves shown in Figure 10 provide information about the future performance of distance estimation methodology; therefore, the errors in distance estimation can be predicted and they will be lower than 4%.

## 4. Experimental Results

In the experimental tests, an SFH4231 IRED [16] and a Basler A622f camera [17] were used. In order to ensure the condition stated in Section 1 the following settings were considered:

- Because the IRED wavelength was 940 nm, the camera was fitted with an interference filter, centered at 940 nm with 10 nm of bandwidth, which was attached to the optic to reduce the influence of background illumination.
- To exclude the influence of the orientation angle, the camera and the IRED were aligned by putting them in a line drawn in the floor. The alignment was obtained by rotating the IRED and the camera using goniometers, until a circular IRED image was obtained. To verify this alignment, several distances between camera and IRED were considered, and in all considered distances the images of the IRED were located in equals image's coordinates.
- Once camera and IRED were put in the correct position and in the considered distance, they were raised from the floor using aluminum bars of squared-section and 1 m height to avoid the reflection on the floor.

Starting with an energetic study, the camera resolution in the radiometric model would not affect the performance. Note that the quantity of energy acquired by the camera will be the same in either a large or a small number of pixels. Evidently, if more pixels could be used, more accurate measurement could be obtained because an average could be used to reduce the spatially distributed noise. Alternatively, in the case of lower resolution cameras, the noise reduction could be achieved by temporal average, which implies to use more images for a single condition.

Currently, small resolutions do not constitute a strict problem from a practical point of view, because most camera sensors have higher than 640 × 480 pixels of resolutions. However, when a higher resolution could be used to estimate the Σ parameter, the result would be more noise-robust. Therefore, we recommend using square-ROI higher than 30 × 30 pixels to guarantee an average with more than 900 pixels. For the experiment performed to validate the distance estimation using the Σ parameter, a 60 × 60 pixels resolution was used.

To validate the standard deviation as an alternative method for estimating the distance between the camera and the IRED, a range of distances from 400 to 800 cm were considered. As shown in Table 1 five distance values for this range were used for calibration purposes as stated in Section 3.1. The measurement process used the entire range of distances; thus some distance values were present which were not used in the calibration process.

**Figure 11.**
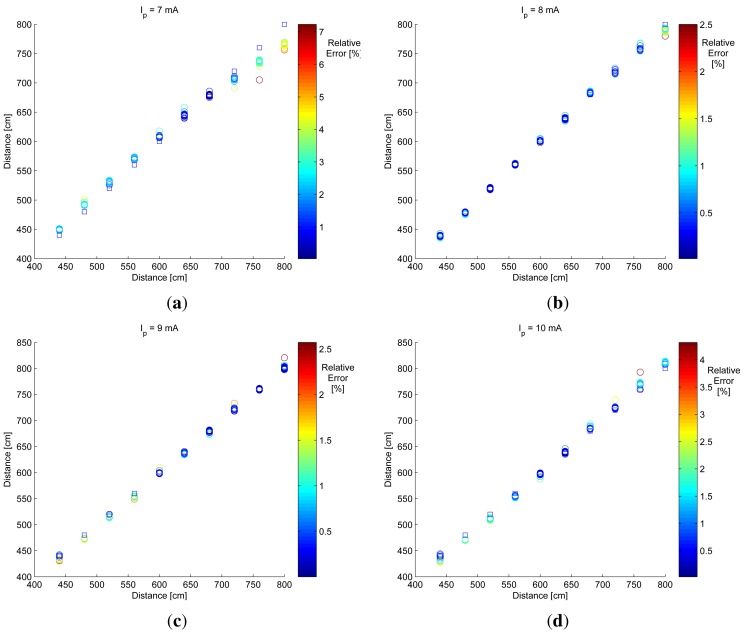
Results of distance estimation process considering all available differences of exposure time for different bias currents: (a) for *I_p_* = 7 mA, (b) for *I_p_* = 8 mA, (c) for *I_p_* = 9 mA and (d) for 10 mA. The line with square markers represents the real distance and the colored circle represents the estimated distance. The color of the circles represents the relative error in distance estimation.

Figure 11 shows the distance estimation considering the differential method defined in Equation (9) for all available exposure time differences (from 1 to 15 ms).

The blue square marker represents the real distance and the colored circles represent the estimated distance. Circle color represents the relative error as a percentage of the distance estimation method. As can be seen from the color of the circles, most relative errors were lower than 5%.

The results shown in Figure 11 were obtained using all available exposure time differences. Thus, if *T* is the total of Δ*t* used for distance measurement, then the differential method will require *T* + 1 images to obtain *T* distance estimations. This implies capturing and processing several images and increases the measurement time.

**Figure 12.**
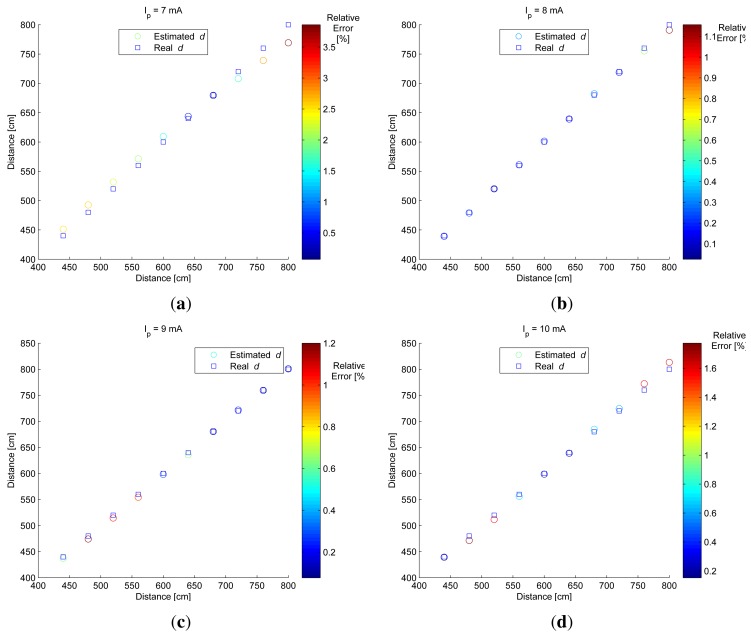
Results of distance estimation process considering the optimum difference of exposure times (Δ*t* = 13 ms) for each of the bias currents used; (a) for *I_p_* = 7 mA, (b) for *I_p_* = 8 mA, (c) for *I_p_* = 9 mA and (d) for 10 mA. The final results are also summarized in Table 2.

A better result and greater efficiency in distance estimation could be obtained if optimum exposure times were used. As stated in Section 3.1 the optimum Δ*t* = 13 ms. Figure 12 shows the results of distance estimation using optimum Δ*t*. Figure 12 is summarized in Table 2.

By using the optimum exposure time difference, the number of images used in the distance estimation process is reduced considerably. In this case, one optimum Δ*t* was used and two images were captured for distance estimation purposes.

As can be seen in Table 2, the relative errors in distance estimation were lower than 3%, therefore; it can be stated that the standard deviation of pixel gray level extracted from the region of interest containing the IRED image is a useful alternative for estimating the distance between the camera and the IRED. In addition, it constitutes an alternative for extracting the depth lost in projective models.

## 5. Conclusions and Future Research

In this paper, we have analyzed the estimation of distance between a camera and an infrared emitter diode. This proposal represents a useful alternative for recovering the depth information lost in projective models.

The alternative proposed in this paper follows the same idea that has been described in the references [10,11], that is: to use only the pixel gray level information of an IRED image to extract depth information.

In addition, in this paper we have demonstrated the need to increase the number of constraints in order to reduce the number of degrees of freedom associated with the problem of estimating camera to emitter distance. The standard deviation alternative proposed here constitutes a helpful alternative.

The modeling process described in this paper was carried out in order to relate the standard deviation to the same magnitudes as those used in [10] and [11]: exposure time, the IRED radiant intensity and the distance between the IRED and the camera, assuming that the camera and the IRED were aligned. These magnitudes were included in the standard deviation model by measuring the individual behaviors of Σ with each of them. From the results of these behaviors, it can be stated that:

- The standard deviation is a linear function of the camera exposure times and IRED radiant intensity.
- The standard deviation is a quadratic function of the inverse-square distance between the camera and the IRED.

By using these conclusions, an expression for standard deviation was derived. The model for standard deviation had 12 coefficients, which were calculated in a calibration process.

The calibration process used images captured with different IRED radiant intensities values, camera exposure times and distances between the camera and the IRED.

By using a differential method, the distance between the camera and the IRED was obtained, using only the pixel gray-level information.

In addition, from data used in the calibration process and considering the differential method, an analysis of model fit was implemented in order to obtain the optimum exposure times to implement the measurement process. The maximum errors of distance estimations considering the optimum times were lower than 3%. Besides, in all experiments carried out to validate the distance estimation method proposed in this paper, the average relative errors were lower than a 1% in the range of distance from 440 to 800 cm.

The goal of this proposal was to define a useful alternative for extracting depth using only pixel gray level information. The main disadvantage of this proposal is that the relationship between the standard deviation and the IRED orientation angle was not considered in the modeling process.

## Figures and Tables

**Table 1.** Data used in the calibration process.

**Magnitude**	**Value**
Distance [cm]	440, 560, 680 and 720
Exposure time [ms]	2, 3, 4, … , 17
IRED's bias current [mA]	7, 8, 9 and 10

**Table 2.** Final distance estimation using the standard deviation of pixel gray-level intensities in an IRED image together with a differential method using the optimum exposure time difference.

**Real distance [cm]**	**Estimated Distance and Relative Errors for Different Bias Currents**							

	***I****_p_* = **7 mA**	**[%]**	***I****_p_* = **8 mA**	**[%]**	***I****_p_* = **9 mA**	**[%]**	***I****_p_* = **10 mA**	**[%]**
440	439.3	0.2	437.3	0.6	439.3	0.2	439.6	0.1
480	478.8	0.3	474.2	1.2	471.5	1.8	468.5	2.4
520	520.1	0.0	514.5	1.1	511.7	1.6	510.2	1.9
560	561.1	0.2	554.4	1.0	556.1	0.7	553.0	1.2
600	601.3	0.2	598.2	0.3	598.6	0.2	597.3	0.4
640	639.1	0.1	635.9	0.6	638.7	0.2	639.0	0.2
680	682.2	0.3	680.5	0.1	684.7	0.7	686.0	0.9
720	718.7	0.2	721.6	0.2	724.4	0.6	728.8	1.2
760	755.4	0.6	759.3	0.1	772.2	1.6	773.6	1.8
800	790.7	1.2	800.8	0.1	813.1	1.6	820.7	2.6
